# Record-low 2025 and 2026 ice extents restore Arctic winter sea-ice decline

**DOI:** 10.1073/pnas.2614134123

**Published:** 2026-07-20

**Authors:** Duo Chan, Alessandro Silvano, Simon A. Josey

**Affiliations:** ^a^ Physical Oceanography, School of Ocean and Earth Science, University of Southampton, Southampton SO14 3ZH, United Kingdom; ^b^ Global Climate Group, National Oceanography Centre, Southampton SO14 3ZH, United Kingdom

**Keywords:** sea ice, Arctic, wintertime, decline, record low

## Abstract

Recent analyses have suggested that the decadal rate of Arctic winter sea-ice extent decline weakened in the early 2020s, with 20-y trends becoming statistically insignificant. Here we show from more up-to-date observations that the exceptionally low 2025 and 2026 ice extent winters reversed this picture, with sea-ice extent during the growth and peak phases returning to record lows and 20-y decline trends becoming significant again. Further analysis of CMIP6 model analogues shows that the observed 2025 decline was unusual but physically plausible under comparable Arctic warming, and is more consistent with ongoing winter sea ice reduction than with a return to values seen in the early 2020s.

Arctic sea ice is a bellwether of high-latitude climate change (1, 2). Since the start of the satellite era, its extent has declined in all seasons (3, 4). Yet over the past two decades, several studies have noted a slowdown in Arctic sea-ice loss, with periods during which 20-y trends weakened or became statistically insignificant (5–8). Such interruptions can arise when internal variability temporarily offsets the forced decline (8).

Although this discussion has focused mainly on the late-summer minimum, a similar picture has emerged in winter (8). Arctic sea-ice extent during the growth and peak phases remained stable through the early 2020s, and the recent winter decline became statistically insignificant. Here we extend the record through 2025 and 2026 and show that the recent winter slowdown was temporary rather than sustained: Sea-ice extent during the growth and peak phases reached record or near-record lows, and the 20-y trend diagnostic again indicates a significant declining trend. Furthermore, analysis of CMIP6 model analogues indicates that the recent observations are more consistent with an ongoing wintertime sea-ice decline than with a return to values observed in the early 2020s.

Arctic sea-ice extent during the 2024–2025 and 2025–2026 seasonal cycles is shown in the context of the full satellite record in Fig. 1*A*. To place the recent evolution within the annual cycle, we divide each August–July cycle into four phases: preconditioning (August–October), growth (November–January), peak (February–March), and decline (April–July). Although neither cycle begins from the lowest late-summer state in the record, both stand out because their sea-ice extent during the growth and peak phases lies largely outside the historical envelope.

**Fig. 1.**
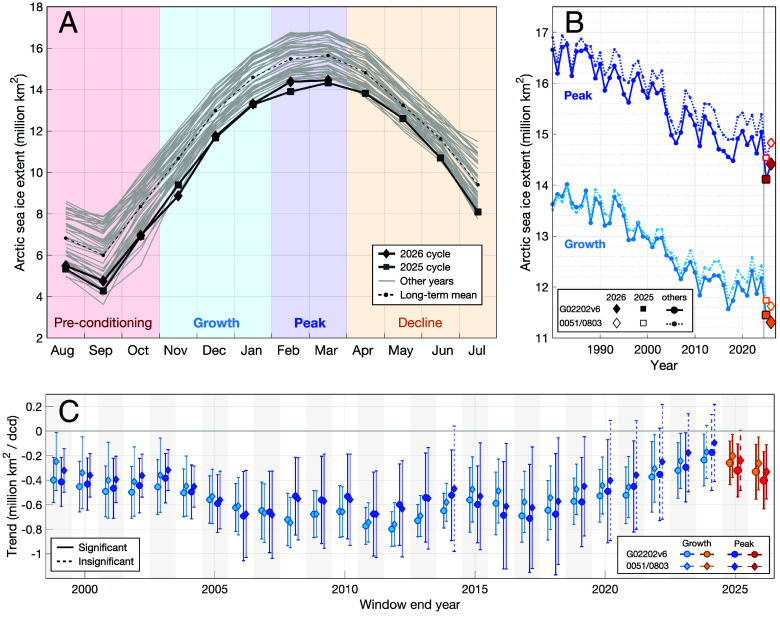
Low Arctic winter sea-ice extent during growth and peak phases in the 2025 and 2026 cycles. (*A*) Monthly Arctic sea-ice extent from August to July for the 2026 cycle (black diamonds) and the 2025 cycle (gray squares), shown against all other years in the observational record (thin gray lines) and the long-term mean seasonal cycle (black dashed line). Shaded backgrounds indicate the four sea-ice phases: preconditioning (August–October), growth (November–January), peak (February–March), and decline (April–July). (*B*) Historical evolution of phase-mean Arctic sea-ice extent during the growth (cyan/orange) and peak (blue/red) phases. Filled symbols show values from the NOAA/NSIDC G02202 v6 product, and open symbols show the sensitivity dataset based on the spliced NSIDC-0051/NSIDC-0803 record. (*C*) Linear trends in Arctic sea-ice extent for the growth (cyan/orange) and peak (blue/red) phases, estimated over moving 20-y windows ending in the year shown on the x-axis. Circles denote estimates from G02202 v6 and diamonds are for the spliced NSIDC-0051/NSIDC-0803 sensitivity dataset. Error bars indicate 95% CI from the AR(1)-based trend model (*SI Appendix*, Extended Methods); solid bars denote trends significant at the 5% level, whereas dashed bars denote insignificant trends.

Averaging sea-ice extent from the primary G02202 v6 record (9); see *SI Appendix*, *Extended Methods*] over each phase makes the exceptional 2025 decline more explicit (filled squares in Fig. 1*B*). After several recent winters with relatively stable growth- and peak-phase extent, the 2025 cycle shows an abrupt drop in both phases, bringing both to record lows at that time. Growth-phase extent fell by 0.72 million km^2^ from 2024 to 2025, equivalent to 5.5% of the 1981–2010 climatological extent, while peak-phase extent fell by 0.93 million km^2^, or 5.8%. These are the largest year-to-year declines in the satellite record for the two phases. Similar declines and record lows are also seen in the spliced NSIDC-0051/NSIDC-0803 sensitivity dataset (10, 11); dashed lines in Fig. 1*B*; see *SI Appendix*, *Extended Methods*].

The 2026 cycle extends this low-winter state (filled diamonds in Fig. 1*B*). During the growth phase, sea-ice extent fell further by 0.14 million km^2^ from 2025 to 2026, reaching a new record low of 11.3 million km^2^. During the peak phase, extent recovered slightly by 0.30 million km^2^ relative to 2025, but still remained the second-lowest value in the satellite record.

The exceptionally low 2025 and 2026 ice extents reverse the recent weakening of Arctic winter sea-ice decline (Fig. 1*C*). We use 20-y sliding windows because this timescale has been central to recent analyses of recent sea-ice slowdown and pause behavior (8, 12). Using this window, the negative trends weaken continuously from 2016 to 2024 in both the growth and peak phases, consistent with previous analyses (7, 8, 12). With the inclusion of the 2025 and 2026 values, however, the declining trends become more negative and return to statistical significance in 2026 in both phases and in both datasets (orange and red in Fig. 1*C*).

To place our observation-based results in a broader climate context, we next compare the observed year-to-year percentage decline in phase-mean sea-ice extent with CMIP6 simulations as a function of corresponding Arctic warming level (Fig. 2 *A* and *B*). This framing reduces the influence of model mean-state biases (13) and allows observational and simulated events to be compared on a common, phase-specific basis (*SI Appendix*, *Extended Methods*). At the observed 2025 Arctic warming level of 2.8 °C (14), the observed growth-phase decline lies near the edge of the CMIP6 95% interval. The peak-phase decline is even more extreme, lying outside the 95% interval but still within the full modeled range. The 2025 event was therefore unusual, but not outside the range of simulated events under comparable Arctic warming.

**Fig. 2.**
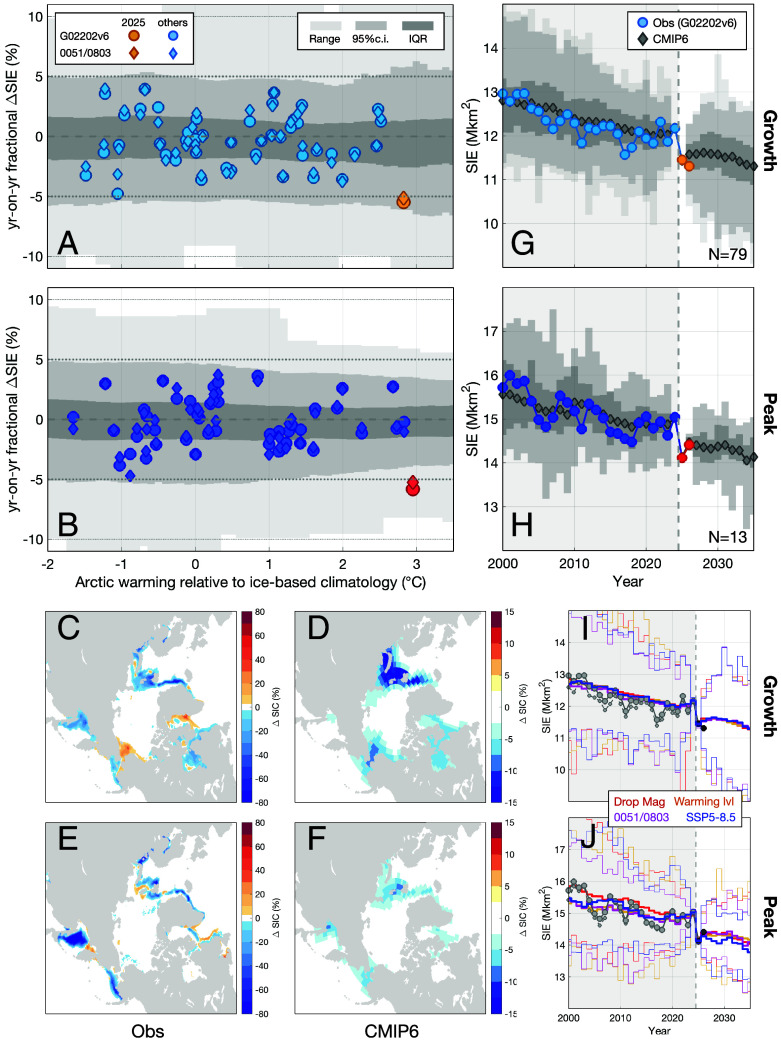
CMIP6-based context for the 2025 winter sea-ice drop and analogue-based post-2025 evolution. (*A*) Year-to-year fractional change in phase-mean Arctic sea-ice extent (ΔSIE; *SI Appendix*, Eq. **3**) vs. corresponding Arctic warming level over the growth phase, shown for G02202 v6 (circles) and the spliced NSIDC-0051/NSIDC-0803 sensitivity dataset (diamonds). The 2025 drop is highlighted in orange. Gray shading shows the CMIP6 distribution, including the full range, 95% interval and interquartile range. (*B*) As in A, but for the peak phase. (*C* and *D*) Year-to-year change in sea-ice concentration (ΔSIC) during the growth phase for the observed 2024–2025 drop (*C*) and the mean composite of the selected CMIP6 analogue events (*D*). (*E* and *F*) As in *C* and *D*, but for the peak phase. Note that left (*C* and *E*) and right (*D* and *F*) panels have different color ranges. (*G*) Observed and analogue-based post-2025 trajectories of phase-mean Arctic sea-ice extent for the growth phase. Circles show observations from G02202 v6 (2025–2026 highlighted in orange), and gray diamonds with shading show the CMIP6 pseudoensemble of conditional post-2025 evolutions (*SI Appendix*, *Extended Methods*), with shading indicating the range, 95% interval and interquartile range, respectively. Numbers indicate the amount of analogue events identified. (*H*) As in *G* but for the peak phase. In panel *H*, the full range is not visible because only 13 events are identified. (*I*) As in *G*, but for the growth phase under the following sensitivity tests: a broadened analogue-selection key of −4% to −7% (red), broader warming-level criterion of 1 to 5°C (orange), the spliced NSIDC-0051/NSIDC-0803 observational dataset (magenta), and SSP5-8.5 (blue). Thin lines mark the full range of CMIP6 analogue projections, and the markers show observations. Those with the spliced NSIDC-0051/NSIDC-0803 dataset are offset by −0.4 million km^2^ for visualization purposes only. (*J*) As in *I*, but for the peak phase.

The observed spatial distribution of the year-to-year sea-ice concentration change in winter 2025 reveals a broad pan-Arctic pattern of negative sea-ice concentration anomalies in both the growth and peak phases (Fig. 2 *C* and *E*). We next use the CMIP6 events as phase-specific analogues to determine whether they exhibit a similar pattern of change and to explore plausible post-2025 evolution (*SI Appendix*, *Extended Methods*). The observed pan-Arctic pattern of ice decline is reproduced in the selected CMIP6 analogue events (Fig. 2 *D* and *F*) although the composite amplitude is weaker because spatially varying internally generated anomalies partially cancel in the model mean.

The analogue trajectories indicate that the 2025 drop is more consistent with subsequent continuation of a low winter sea-ice state than with a rebound to the values seen in the early 2020s (Fig. 2 *G* and *H*). For the growth phase, the pseudoensemble spans a relatively wide range of post-2025 outcomes, reflecting larger internal variability, but remains centered on continued low extent. The observed 2026 value falls within this spread, consistent with a further downward step after 2025. For the peak phase, the analogue response is more coherent: all identified events show a modest rebound in the following year, but not a return to pre-2025 conditions. The observed partial recovery in 2026 is consistent with this behavior, while the analogue mean over subsequent years remains well below the pre-2025 level. These results are robust to alternative analogue-selection thresholds, warming-level criteria, observational datasets, and forcing scenarios (Fig. 2 *I* and *J*).

As an additional check, we extended the sliding-window trend calculation along the CMIP6 analogue trajectories. In the core analysis setup, the analogue-conditioned probability that the 20-y peak-phase extent trend remains significantly negative stays above 0.75 through 2030, indicating that the restored significance is unlikely to be immediately reversed, at least in the analogue ensemble.

Together, these results show that the exceptionally low 2025 and 2026 winters materially alter the observational picture of recent Arctic winter sea-ice evolution. The extended record no longer supports a sustained pause; the 20-y trend diagnostic returns to significant decline, providing observational evidence consistent with the interpretation that internal variability temporarily offset the forced decline (8). CMIP6 analogues further indicate that the 2025 drop is more consistent with continued low winter sea-ice extent than with a rapid rebound. This behavior is consistent with continued forced decline limiting recovery from a large internally generated drop. Our 20-y trend finding points to a decadal modulation of ongoing winter ice loss rather than an interruption of the longer-term decline. This is consistent with a longer timescale 30-y trend analysis that we have also carried out which shows uninterrupted decline. It also motivates further study of how winter ice loss is reshaping the Arctic seasonal cycle and its capacity for recovery.

## Materials and Methods

Sea-ice concentrations are from the NOAA/NSIDC Climate Data Record of Passive Microwave Sea-Ice Concentration, Version 6, as well as NSIDC-0051 Version 2 (10) and NSIDC-0803 Version 2 (11). Please see *SI Appendix*, *Extended Methods* for further methodological information.

## Supplementary Material

Appendix 01 (PDF)

## Data Availability

The following data products are publicly available: NSIDC-G02202 v6 (9), NSIDC-0051 (10), and NSIDC-0803 (11); CMIP6 (15); and DCENT-I (16). Code and processed data for replication are available at https://doi.org/10.7910/DVN/40JUN6 (17).
