# Supplementary material for: Record-low 2025 and 2026 ice extents restore Arctic winter sea-ice decline
Source: Proc Natl Acad Sci U S A. 2026 Jul 20;123(30):e2614134123. doi: 10.1073/pnas.2614134123 (PMC13415921; doi:10.1073/pnas.2614134123)
Supplement: Supplementary file 1 — Appendix 01 (PDF) [file pnas.2614134123.sapp.pdf]

# Supporting Information for

## Record-low 2025 and 2026 ice extents restore Arctic winter sea-ice decline

**Duo Chan, Alessandro Silvano, Simon A. Josey**

### Extended Methods

**Sea-ice concentration and extent.** As the primary dataset, we use the NOAA/NSIDC Climate Data Record of Passive Microwave Sea-Ice Concentration, Version 6 (G02202 v6), developed for long-term, consistent, and reproducible sea-ice concentration analyses (1). As a sensitivity test, we additionally construct a spliced daily concentration record using NSIDC-0051 Version 2 for 1979–2025 (2) and NSIDC-0803 Version 2 for 2026 (3). Because this auxiliary record spans a product transition, we treat it as a sensitivity dataset rather than as a single homogeneous climate data record. Both NSIDC-0051 and NSIDC-0803 contain an unobserved region near the North Pole (the pole hole). For the sensitivity analysis only, missing values in this region are filled using the corresponding G02202 v6 values to avoid artificial discontinuities in extent calculations. Because our sensitivity analysis is restricted to November–March, when the Arctic sea-ice edge lies far south of the pole-hole region, this treatment is not expected to materially affect hemispheric sea-ice extent.

Monthly SIE in the main analysis is calculated from monthly mean sea-ice concentration fields, defined as the sum of grid-cell areas with sea-ice concentration $\geq 15\%$. We note that the recommended procedure for observational monthly SIE is to compute daily SIE from daily SIC and then average the daily SIE values to monthly means. Yet our choice here is to maintain consistency with the monthly CMIP6 SIC fields used in the analogue analysis (see below). Repeating the observational trend and record-low analyses using this daily-SIE-first procedure produces a small mean offset in SIE but does not affect the severity of the 2025 growth- and peak-phase drops, the record or near-record status of the 2025–2026 winter extents, or the restoration of significant negative 20-year trends.

**Calculating linear trends in sea-ice extent.** To estimate linear trends while accounting for serial correlation in the residuals, we model monthly sea-ice extent as a linear function of time with autoregressive errors of order 1 [AR(1)]:

$$y_t = kt + b + \varepsilon_t, \qquad [1]$$

$$\varepsilon_t = \phi\varepsilon_{t-1} + u_t, \qquad [2]$$

where $y_t$ is monthly sea-ice extent at time step $t$, $k$ is the linear trend, $b$ is the intercept, $\phi$ is the lag-1 autocorrelation coefficient, and $u_t$ is a white-noise error term. The model is estimated using MATLAB's `regARIMA` framework (4). The trend, intercept, AR(1) coefficient and innovation variance are estimated jointly by maximum likelihood. Regression models with AR(1) errors are widely used for estimating climate trends and their uncertainties when residuals are serially correlated (5, 6). Compared with ordinary least squares, this approach provides more reliable uncertainty estimates when errors are serially correlated. The estimated AR(1) coefficient varies across windows and phases. For the growth phase, the estimated $\phi$ values range from -0.08 to 0.35, with a mean of 0.14. For the peak phase, the estimated $\phi$ values range from -0.03 to 0.53, with a mean of 0.31. Thus, residual autocorrelation is generally weak to moderate in the growth phase and more frequently positive in the peak phase.

Ninety-five percent confidence intervals for the linear trend are computed as $\hat{k} \pm 1.96\,\mathrm{SE}(\hat{k})$, where $\hat{k}$ is the estimated trend and $\mathrm{SE}(\hat{k})$ is its standard error from the fitted model. As a diagnostic check on the normal-error assumption used for confidence intervals, we tested the standardized, pre-whitened residuals ($u_t$ in equation 2) from each 20-year AR(1) trend model using a Kolmogorov–Smirnov test (7). No 20-year window rejects the Gaussian-error assumption at the 5% significance level for either the growth or peak phase.

**CMIP6 models.** We use monthly surface air temperature (tas) and sea-ice concentration (siconc) from the historical (1850–2014) and SSP2-4.5 (2015–2100) experiments from 20 CMIP6 models (8): ACCESS-CM2 (3 members), BCC-CSM2-MR (1), CAMS-CSM1-0 (2), CanESM5 (25), CESM2-WACCM (3), CMCC-CM2-SR5 (1), CMCC-ESM2 (1), EC-Earth3 (22), EC-Earth3-CC (1), EC-Earth3-Veg-LR (3), FIO-ESM-2-0 (3), GFDL-ESM4 (3), INM-CM4-8 (1), INM-CM5-0 (1), IPSL-CM6A-LR (11), MIROC6 (3), MPI-ESM1-2-HR (2), MPI-ESM1-2-LR (10), NESM3 (2), and NorESM2-MM (2). SSP2-4.5 refers to the Shared Socioeconomic Pathway (9) with 4.5 W/m$^2$ of forcing by 2100. For each ensemble member, the historical and SSP2-4.5 simulations are concatenated into a continuous 1850–2100 time series. All available members on Google Cloud via the Pangeo Project are used (10). As a sensitivity test, SSP5-8.5 (a higher emission scenario with 8.5 W/m$^2$ of forcing by 2100) simulations are also analysed.

**Climatological period.** For observational datasets, the reference period is 1981–2010, following community convention (e.g., ref. (11)). For CMIP6 simulations, given that models differ in their climatological sea-ice states (12), we define a simulation-specific climatological reference period based on each model's sea-ice extent. Specifically, for each simulation and for each sea-ice phase considered (pre-conditioning: August–October of the previous year; growth: November of the previous year to January of the current year; peak: February–March; decline: April–July), we compute a 31-year running mean of phase-mean sea-ice extent and identify the 31-year window whose mean extent is closest to the corresponding observed 1981–2010 phase-mean

extent derived from G02202 v6. This approach is intended to reduce the influence of inter-model biases in climatological sea-ice mean state when comparing year-to-year fractional changes in sea-ice extent across models and against observations. We define the year-to-year fractional change as

$$\Delta_t = \frac{\mathrm{SIE}_t - \mathrm{SIE}_{t-1}}{\overline{\mathrm{SIE}}}, \qquad [3]$$

where $\mathrm{SIE}_t$ is phase-mean sea-ice extent in year $t$, and $\overline{\mathrm{SIE}}$ is the climatological mean sea-ice extent for the corresponding phase-specific reference period.

For contextual comparison, Arctic warming level is defined as the area- weighted mean of near-surface air temperature anomaly (tas) north of 60°N relative to the corresponding climatological mean over the reference period for each model in its respective phase. For observations, surface temperature is taken from DCENT-I (13), and the reference period is 1981–2010.

**Post-2025 evolution using CMIP6 analogues.** To assess plausible post-2025 Arctic winter sea-ice evolution, we perform a phase-specific analogue search in CMIP6 simulations. For each sea-ice phase considered, candidate analogue events are identified as model years with year-to-year fractional declines in sea-ice extent between 5% and 6% (Eq. 3) and phase-consistent Arctic warming levels between 2°C and 4°C.

For each identified analogue event, the subsequent model trajectory is re-expressed on the observed extent scale by multiplying the modelled fractional changes using the observed climatological extent for the corresponding phase. These de-normalised changes are then cumulatively summed forward from the aligned event year to reconstruct the analogue extent trajectory. The resulting trajectory is then shifted so that the mean sea-ice extent over the two analogue years spanning the identified drop (i.e., the year before the drop and the drop year itself) matches the observed mean over 2024–2025 for that phase. The subsequent ten annual phase-mean SIE values, starting from the first year after the drop, are then appended to the observed sea-ice extent record after 2025, corresponding to a conditional post-2025 trajectory for 2026–2035. Repeating this procedure for all identified analogue events yields a pseudo-ensemble of conditional post-2025 evolutions comparable with the observed 2025 event. Because the analogue selection is based on normalised change, the pseudo-ensemble should be interpreted as a set of statistically plausible conditional trajectories rather than as a physics-based deterministic forecast.

Sensitivity tests are performed by varying the analogue-selection thresholds to year-to-year fractional declines between 4% and 7% and phase-consistent Arctic warming levels between 1°C and 5°C. We also repeat the analogue construction using the spliced NSIDC-0051/NSIDC-0803 record as the observational reference, with analogue trajectories appended to that record after 2025 in the same manner. The analogue analysis is further repeated under SSP5-8.5 as an additional sensitivity test.


1. WN Meier, F Fetterer, AK Windnagel, JS Stewart, T Stafford, Noaa/nsidc climate data record of passive microwave sea ice concentration (2026).
2. N DiGirolamo, CL Parkinson, DJ Cavalieri, P Gloersen, HJ Zwally, Sea ice concentrations from nimbus-7 smmr and dmsp ssm/i-ssmis passive microwave data (2022).
3. JS Stewart, WN Meier, R Marowitz, DJ Scott, H Wilcox, Amsr2 daily polar gridded sea ice concentrations (2025).
4. The MathWorks, Inc., Econometrics toolbox (2026).
5. BD Santer, et al., Statistical significance of trends and trend differences in layer-average atmospheric temperature time series. *J. Geophys. Res. Atmospheres* **105**, 7337–7356 (2000).
6. C Proistosescu, PJ Huybers, Slow climate mode reconciles historical and model-based estimates of climate sensitivity. *Sci. Adv.* **3**, e1602821 (2017).
7. FJ Massey Jr, The kolmogorov-smirnov test for goodness of fit. *J. Am. statistical Assoc.* **46**, 68–78 (1951).
8. V Eyring, et al., Overview of the Coupled Model Intercomparison Project Phase 6 (CMIP6) experimental design and organization. *Geosci. Model. Dev.* **9**, 1937–1958 (2016).
9. K Riahi, et al., The shared socioeconomic pathways and their energy, land use, and greenhouse gas emissions implications: An overview. *Glob. environmental change* **42**, 153–168 (2017).
10. RP Abernathey, et al., Cloud-native repositories for big scientific data. *Comput. Sci. & Eng.* **23**, 26–35 (2021).
11. MC Serreze, J Stroeve, Arctic sea ice trends, variability and implications for seasonal ice forecasting. *Philos. Transactions Royal Soc. A: Math. Phys. Eng. Sci.* **373**, 20140159 (2015).
12. D Notz, S Community, Arctic sea ice in CMIP6. *Geophys. Res. Lett.* **47**, e2019GL086749 (2020).
13. D Chan, et al., DCENT-I: A globally infilled extension of the dynamically consistent ensemble of temperature dataset. *Geosci. Data J.* **13**, e70054 (2026).
